# Schistosomiasis: an unexpected finding in appendicitis

**DOI:** 10.1093/jscr/rjae727

**Published:** 2025-02-17

**Authors:** Jhanvi Dholakia, Maiko Smith

**Affiliations:** Department of General Surgery, Waikato Hospital, 183 Pembroke Street, Hamilton 3200, New Zealand; Department of General Surgery, Waikato Hospital, 183 Pembroke Street, Hamilton 3200, New Zealand

**Keywords:** schistosomiasis, appendicitis, Schistosoma infection

## Abstract

Schistosomiasis is an uncommon cause of appendicitis in the developed world. It needs to be correctly identified, as it has further treatment implications for the patient. We present the case of a 34-year-old Zimbabwean migrant with appendicitis and associated adjacent peritoneal nodules. Histological testing showed these to be parasitic larvae, Schistosoma. He was followed up by the infectious diseases team and treated with Praziquantel as eradicative therapy. Appendicitis may be the only sign of a Schistosoma infection, which would enable early eradicative treatment, thus preventing the development of more serious hepato-intestinal complications.

## Introduction

Schistosomiasis is parasitic disease caused by trematode worms. It is prevalent in tropical in countries where inhabitants are exposed to poor sanitation, and commonly affects the urogenital and intestinal system [1]. When it affects the intestinal system, it usually causes diarrhoea, dyspepsia, and malnutrition, however, appendicitis is uncommon. Appendicitis is the most common intra-abdominal surgical pathology universally and accounts for 5000 admissions in New Zealand every year [2]. In the Western world, infections, neoplasms and appendicoliths are common causes of appendicitis, but parasitic infection resulting in appendicitis is rare [3]. However, it is important that parasitic infections are considered, as patients may need further medical treatment.

## Case report

A 34-year-old Zimbabwean man presented to a New Zealand hospital with acute onset right lower quadrant pain, worsening over 2 days, which was not associated with any gastrointestinal or genitourinary symptoms. He had an associated elevation in his inflammatory markers. He had no comorbidities and took no regular medications. A detailed history found that he had lived in Zimbabwe until 13 years of age before migrating to New Zealand, and he has lived here since.

He was thought to have clinical appendicitis and underwent a laparoscopic appendicectomy. Intraoperatively, the appendix didn’t look overtly inflamed, but the surgeon noted nodules overlying the appendix, the paracolic gutter and in the rectovesical pouch. Some of these nodules were excised along with the appendix to rule out a disseminated intraperitoneal appendiceal neoplasm.

He made an uneventful recovery and was discharged on Day 2.

Histopathology showed faecal impaction within the appendix and lymphoid hyperplasia. The nodules showed dystrophic calcification and chitinous material within, which was thought to represent parasitic larvae consistent with Schistosomiasis. His serological testing for Schistosomiasis was inconclusive.

Subsequently, he was reviewed by the Infectious Diseases department at our hospital and treated with Praziquantel as eradicative therapy, given there was intraperitoneal spread.

## Discussion

Schistosomiasis is a parasitic disease endemic to large areas of sub-Saharan Africa and *Schistosoma mansoni* and *Schistosoma japonica* are the two most common parasites involved in gastrointestinal infections [4]. It affects rural populations and lower socioeconomic groups, specifically agriculture and fishing populations where poor sanitation may be prevalent. In endemic countries such as Nigeria, a retrospective review found 4.2% of resected appendices between 1991 and 2004 had Schistosoma ova on histology [5]. However, Schistosoma infections are very rare in developed countries [6].

The exact pathophysiology of appendicitis with Schistosomiasis is unknown. Terada describes egg emboli causing ischemia of the appendix and a subsequent drop in mucosal immunity which causes bacterial translocation and infection leading to acute appendicitis [6].

The World Health Organization recommends Praziquantel, an anthelminthic, as treatment for Schistosoma infections, with repeated treatments in young children from endemic areas to reduce the risk of developing chronic infections [1]. A systematic review regarding treatment post Schistosomiasis highlighted the variability in treatment worldwide, with 38% of the patients being treated with alternate regimes such as higher doses, longer durations, and repeated treatments with Praziquantel [7].

Acute Schistosomiasis may be asymptomatic, however, chronic infections can lead to hepato-intestinal complications, such as hypoalbuminemia, portal hypertension, ascites and variceal bleeding that are associated with significant morbidity and mortality. Appendicitis may be the only early manifestation of an infection and therefore it is prudent to identify the causative agent correctly, so that eradicative treatment can be prescribed. This case calls attention to the importance of questioning patients about their travel history, associated risk factors and including unexpected causes of appendicitis in the differential diagnosis.

## Conclusion

Schistosomiasis is a rare but significant finding as a cause of appendicitis. It should be appropriately followed up with Praziquantel treatment when identified due to the potential.

## Conflict of interest statement

None declared.

## Funding

None declared.
